# Thrombomodulin is upregulated in the kidneys of women with pre-eclampsia

**DOI:** 10.1038/s41598-021-85040-9

**Published:** 2021-03-11

**Authors:** Cleo C. L. van Aanhold, Manon Bos, Katrina M. Mirabito Colafella, Marie-Louise P. van der Hoorn, Ron Wolterbeek, Jan A. Bruijn, Kitty W. M. Bloemenkamp, Anton H. van den Meiracker, A. H. Jan Danser, Hans J. Baelde

**Affiliations:** 1 Department of Pathology, Leiden University Medical Center, L1Q, Room P0-107, 2300 RC Leiden, The Netherlands; 2 Department of Obstetrics and Gynaecology, Leiden University Medical Center, Leiden, The Netherlands; 3 Department of Internal Medicine, Erasmus Medical Center, Rotterdam, The Netherlands; 4 Cardiovascular Disease Program, Biomedicine Discovery Institute and Department of Physiology, Monash University, Melbourne, Australia; 5 Department of Biomedical Data Sciences, Leiden University Medical Center, Leiden, The Netherlands; 6 Department of Obstetrics, Birth Center, University Medical Center Utrecht, Utrecht, The Netherlands

**Keywords:** Kidney diseases, Pathogenesis, Translational research

## Abstract

The endothelial glycoprotein thrombomodulin regulates coagulation, vascular inflammation and apoptosis. In the kidney, thrombomodulin protects the glomerular filtration barrier by eliciting crosstalk between the glomerular endothelium and podocytes. Several glomerular pathologies are characterized by a loss of glomerular thrombomodulin. In women with pre-eclampsia, serum levels of soluble thrombomodulin are increased, possibly reflecting a loss from the glomerular endothelium. We set out to investigate whether thrombomodulin expression is decreased in the kidneys of women with pre-eclampsia and rats exposed to an angiogenesis inhibitor. Thrombomodulin expression was examined using immunohistochemistry and qPCR in renal autopsy tissues collected from 11 pre-eclamptic women, 22 pregnant controls and 11 hypertensive non-pregnant women. Further, kidneys from rats treated with increasing doses of sunitinib or sunitinib in combination with endothelin receptor antagonists were studied. Glomerular thrombomodulin protein levels were increased in the kidneys of women with pre-eclampsia. In parallel, in rats exposed to sunitinib, glomerular thrombomodulin was upregulated in a dose-dependent manner, and the upregulation of glomerular thrombomodulin preceded the onset of histopathological changes. Selective ET_A_R blockade, but not dual ET_A/B_R blockade, normalised the sunitinib-induced increase in thrombomodulin expression and albuminuria. We propose that glomerular thrombomodulin expression increases at an early stage of renal damage induced by antiangiogenic conditions. The upregulation of this nephroprotective protein in glomerular endothelial cells might serve as a mechanism to protect the glomerular filtration barrier in pre-eclampsia.

## Introduction

Pre-eclampsia affects 3–5% of pregnant women and is an important cause of maternal and neonatal morbidity and mortality^1^. Pre-eclampsia is characterised by placenta dysfunction and placental production of several antiangiogenic and pro-inflammatory factors (e.g. soluble Flt-1, soluble endoglin and TNF-alpha), which contribute to systemic endothelial dysfunction, an increase in vascular resistance and complications in multiple organs^2^. The kidney is frequently affected in women with pre-eclampsia, with symptoms ranging from mild proteinuria to nephrotic-range proteinuria and kidney failure at later stages of the disease^3^. The pathological changes in the kidneys of women with pre-eclampsia are characterised by endotheliosis, podocyte foot process effacement and podocyte loss^4,5^. What drives these pathological changes is not fully understood.

Renal dysfunction in pre-eclampsia is likely caused by a disruption of signalling pathways involved in maintaining the glomerular filtration barrier^6^. For instance, increased levels of the antiangiogenic factor soluble Flt-1 lead to impaired VEGF signalling in the glomerulus, resulting in a disruption of the glomerular filtration barrier, proteinuria and glomerular endotheliosis^7,8^. In accordance, drugs that inhibit angiogenic signalling such as sunitinib are associated with adverse effects that mimic the pre-eclampsia kidney phenotype^9^. Another important mediator of hypertension and renal injury in pre-eclampsia and VEGF inhibition is the endothelin system^10^. Endothelin is a potent vasoconstrictor produced by endothelial cells which signals through the endothelin type A receptor (ET_A_R) on vascular smooth muscle cells, resulting in vasoconstriction^11^. Conversely, endothelin signalling through the endothelin type B receptor (ET_B_R) results in production of nitric oxide and prostacyclin which promote vasodilatation^11,12^. ETR blockade, and selective ET_A_R antagonism in particular, effectively reduces blood pressure and proteinuria in rodent models of pre-eclampsia and VEGF-inhibition^13^. Similarly, we recently demonstrated that selective ET_A_R blockade rescues the renal phenotype of rats exposed to VEGF-inhibition^14^.

Thrombomodulin is a transmembrane glycoprotein expressed primarily by endothelial cells, and is a component of the endothelial surface glycocalyx^15^. Thrombomodulin maintains vascular homeostasis by regulating coagulation, inflammation and apoptosis^15,16^. In women with pre-eclampsia, increased serum levels of soluble thrombomodulin have been reported^17^, which likely reflect increased cleavage of the thrombomodulin ectodomain from the endothelium^18^. Thrombomodulin plays a pivotal role in the maintenance of the glomerular filtration barrier in diabetic mice: perturbed thrombomodulin signalling leads to increased apoptosis of glomerular endothelial cells and podocytes and increased glomerular complement activation, resulting in aggravated proteinuria and glomerulosclerosis^19,20^. Thus, thrombomodulin has important cytoprotective effects on the glomerular filtration barrier and a disruption of thrombomodulin signalling may be involved in kidney disease in pre-eclampsia.

In this study, we investigated whether thrombomodulin expression is decreased in the kidneys of women with pre-eclampsia and in a rat model of VEGF-inhibition. Furthermore, we investigated the role of the endothelin system on renal thrombomodulin expression in this rat model by using a selective ET_A_R antagonist and a dual ET_A/B_R antagonist.

## Methods

### Patient material

The renal autopsy cohort used in this study has been described before (see:^21^). In short, a nationwide search of the Dutch Pathology Registry (PALGA) was conducted to collect renal tissues from patients who were pregnant and were confirmed cases of pre-eclampsia. In addition, two control groups were included: (1) pregnant women without a hypertensive disorder prior to or during pregnancy and (2) young non-pregnant women with a medical history of chronic hypertension. The pathology data were linked with the records of the National Maternal Mortality Committee of the Dutch Society of Obstetrics. Pre-eclampsia was defined based on the diagnostic criteria of the International Society for the Study of Hypertension in Pregnancy (ISSHP)^22^. Paraffin-embedded kidney samples from 11 women with pre-eclampsia, 22 normotensive pregnant controls and 11 non-pregnant hypertensive controls were available for this study. All autopsy samples were coded and then handled and analysed anonymously in accordance with the Dutch national ethics guidelines (Code for Proper Secondary Use of Human Tissue, Dutch Federation of Medical Scientific Societies). This study was approved by the Leiden University Medical Center Medical Ethics Committee (license number P12.107).

### Rats

Experiments were performed in accordance with the guidelines from Directive 2010/63/EU of the European Parliament and the Netherlands Experiments on Animals Act, after obtaining approval from the Erasmus Medical Center Animal Ethics Committee (license number 118-16-01). Male Wistar Kyoto rats (WKY, 280–300 g) were obtained at 10 weeks of age. The animals were housed in an experimental room with temperature maintained at 21–22 °C and a 12-h light/dark cycle. Animals had access to standard laboratory rat chow and water ad libitum. Two different experiments were used in this study, with detailed methods and the phenotype of these animals published previously^14,23^. In brief, rats were treated with different doses of sunitinib with or without ETR antagonists for 8 days. Aortic blood pressure was measured using radiotelemetry. Before and after administration of the treatment(s), rats were housed in metabolic cages for 48 h. The first day was used for acclimatization and the second day for the collection of 24-h urine samples. In the first study, rats were randomly assigned to receive a low, intermediate or high dose of sunitinib (7, 14 or 26.7 mg/kg/day p.o. sunitinib-L-malate (Sutent, Pfizer), respectively) or vehicle for 8 days. At the end of the experiment, rats were euthanised with 60 mg/kg pentobarbital intraperitoneal and exsanguination via abdominal vein puncture^23^. In the second study, rats were administered vehicle or the intermediate dose of sunitinib (14 mg/kg/day p.o.) alone or in combination with macitentan (dual ET_A/B_R antagonist, 100 mg/kg/day p.o.) or sitaxentan (selective ET_A_R antagonist, 30 or 100 mg/kg/day p.o.). At the end of the experiment, rats were euthanised by via Forane (isoflurane) anaesthesia overdose and exsanguination via abdominal vein puncture^14^. In both studies, following euthanasia, the kidneys were rapidly excised for subsequent analyses.

### Renal histology

Kidney sections were blindly evaluated by a pathologist for the presence or absence of endothelial cell swelling, epithelial cell swelling, ischemia and intra-epithelial protein. For electron microscopy two glomeruli were examined, the presence of glomerular endotheliosis and podocyte morphology was studied. Histopathological findings in human subjects and animals exposed to different concentrations of sunitinib have been published before^21,23^.

### Immunohistochemistry

Kidney sections were deparaffinised. Heat-induced antigen retrieval was performed using citrate (anti-human thrombomodulin antibody) or Tris/EDTA (anti-rat thrombomodulin and anti-ET_A_R antibodies) buffers. Peroxidase was blocked in a hydrogen peroxide solution for 20 min. Kidney samples were incubated with a mouse monoclonal anti-human thrombomodulin (1:200, Leica Biosystems, Danvers), a rabbit monoclonal anti-mouse/rat thrombomodulin (1:2000, Abcam) or a rabbit polyclonal anti-ET_A_R (1:700, Invitrogen, Carlsbad) antibody for one hour at room temperature. Binding of the primary antibody was visualised with peroxidase-labelled anti-mouse or labelled anti-rabbit polymer (Dako, Denmark) and diaminobenzidine as a chromogen.

### Staining analysis

Thrombomodulin and ET_A_R staining were scored by two independent observers blinded with respect to clinical diagnosis and treatment (CCLA and MB). Twenty-five randomly selected glomeruli per sample were scored using a semi-quantitative scale as follows: 0, absent; 1, < 10% glomerular staining; 2, 10–49% glomerular staining; 3, 50–90% of glomerular staining; and 4, > 90% of glomerular staining. Representative examples of the different scores are shown in Supplementary Figures S1, S2, S3. Interobserver agreement was minimally sufficient in all scoring experiments (κ ≥ 0.5). When observers scored glomeruli differently, consensus was obtained.

### qPCR

Quantitative PCR was performed to quantify mRNA expression of thrombomodulin and ET_A_R. RNA was isolated with TRIzol reagent (Lifetechnologies, San Francisco, CA, USA). Synthesis of cDNA was performed with AMV reverse transcriptase (Roche, Basel, Switzerland), and SYBR Green quantitative PCR was performed according to the manufacturer’s protocol (Bio-Rad Laboratories Inc, Hercules, CA, USA). Primer sequences are described in Supplementary Table S1. Expression was measured by the comparative threshold cycle method and normalised to hypoxanthine phosphoribosyltransferase expression. A melting curve analysis was performed to verify the specificity of amplification.

### Statistical analysis

Interobserver variation was determined using the kappa statistic. Normally distributed continuous data were analysed using one-way ANOVA followed by the least significant difference post-hoc test for the planned pairwise group comparisons. To analyze differences in glomerular thrombomodulin expression scores between groups in the autopsy cohort, ordinal logistic regression for clustered data with generalized estimating equations (GEE) was used. To analyse putative correlations between thrombomodulin expression and histopathological or clinical findings, Pearson’s correlation was used. The shapes of the thrombomodulin and ET_A_R response curves over the sunitinib dose range were examined by means of regression lines. *p* < 0.05 was considered statistically significant. Unless stated otherwise, summary data in the figures are reported as the mean ± SD.

## Results

### Glomerular thrombomodulin is increased in the kidneys of women with pre-eclampsia

Thrombomodulin staining was analysed in kidney samples from 11 women with pre-eclampsia, 22 normotensive pregnant controls and 11 non-pregnant hypertensive controls. The hypertensive controls were older than women of the other 2 study groups; compared to pregnant controls, pre-eclamptic women had longer gestation and higher blood pressure (Supplemental Table S2).

**Figure 1:**
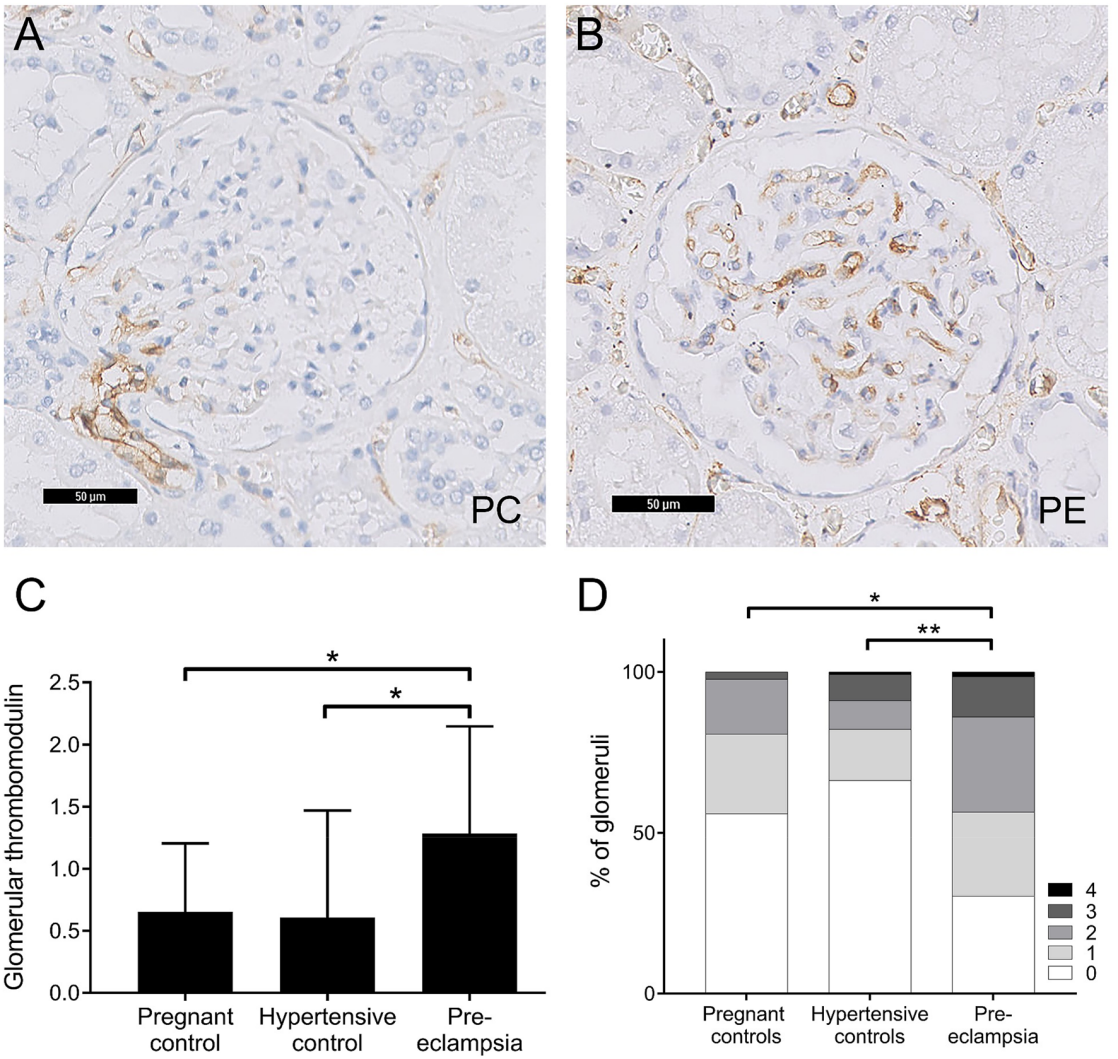
Glomerular thrombomodulin protein is increased in the kidneys of women with pre-eclampsia. (**a**) Representative example of glomerular thrombomodulin staining in a non-hypertensive pregnant control. (**b**) Representative example of glomerular thrombomodulin staining in a pre-eclampsia case. (**c**) Averaged glomerular thrombomodulin protein scores in women with pre-eclampsia (n = 11), pregnant controls (n = 22) and non-pregnant hypertensive controls (n = 11). (**d**) Distribution of the glomerular thrombomodulin protein scores in the indicated groups; **p < 0.01 and *p < 0.05.

Regardless of the presence of thrombomodulin in the glomerulus, thrombomodulin was present in the peritubular capillaries in all studied samples (Supplemental Figure S1). In glomeruli, thrombomodulin expression seemed to originate from the vascular pole (Fig. 1A). On average, glomerular thrombomodulin expression was higher in the pre-eclamptic patients compared to pregnant and hypertensive controls (Fig. 1); compared to pre-eclampsia, controls had lower glomerular thrombomodulin expression scores (pregnant controls, OR: 3.0; 95%-CI: (1.1, 7.8), *p* < 0.05; and hypertensive controls, OR: 6.1; 95%-CI: (1.6, 23.3), *p* < 0.01). In pre-eclampsia cases, glomerular thrombomodulin protein levels were inversely associated with the presence of glomerular endotheliosis (r: − 0.71; *p* < 0.05). Furthermore, in hypertensive controls, thrombomodulin levels were inversely associated with the glomerular tuft size (r: − 0.79, *p* < 0.01).

### Glomerular thrombomodulin expression in rats exposed to sunitinib

To better understand our findings in pre-eclamptic kidneys, glomerular thrombomodulin staining was studied in rats exposed to increasing doses of the VEGF-receptor inhibitor sunitinib. Administration of sunitinib resulted in an increase in blood pressure and proteinuria in a dose-dependent manner^23^. Rats exposed to the low dose of sunitinib had no histopathological anomalies; endotheliosis was observed in the kidneys of rats exposed to the intermediate and high doses of sunitinib; fibrin deposits were only seen in the glomerular capillaries of animals exposed to a high dose of sunitinib^23^.

**Figure 2:**
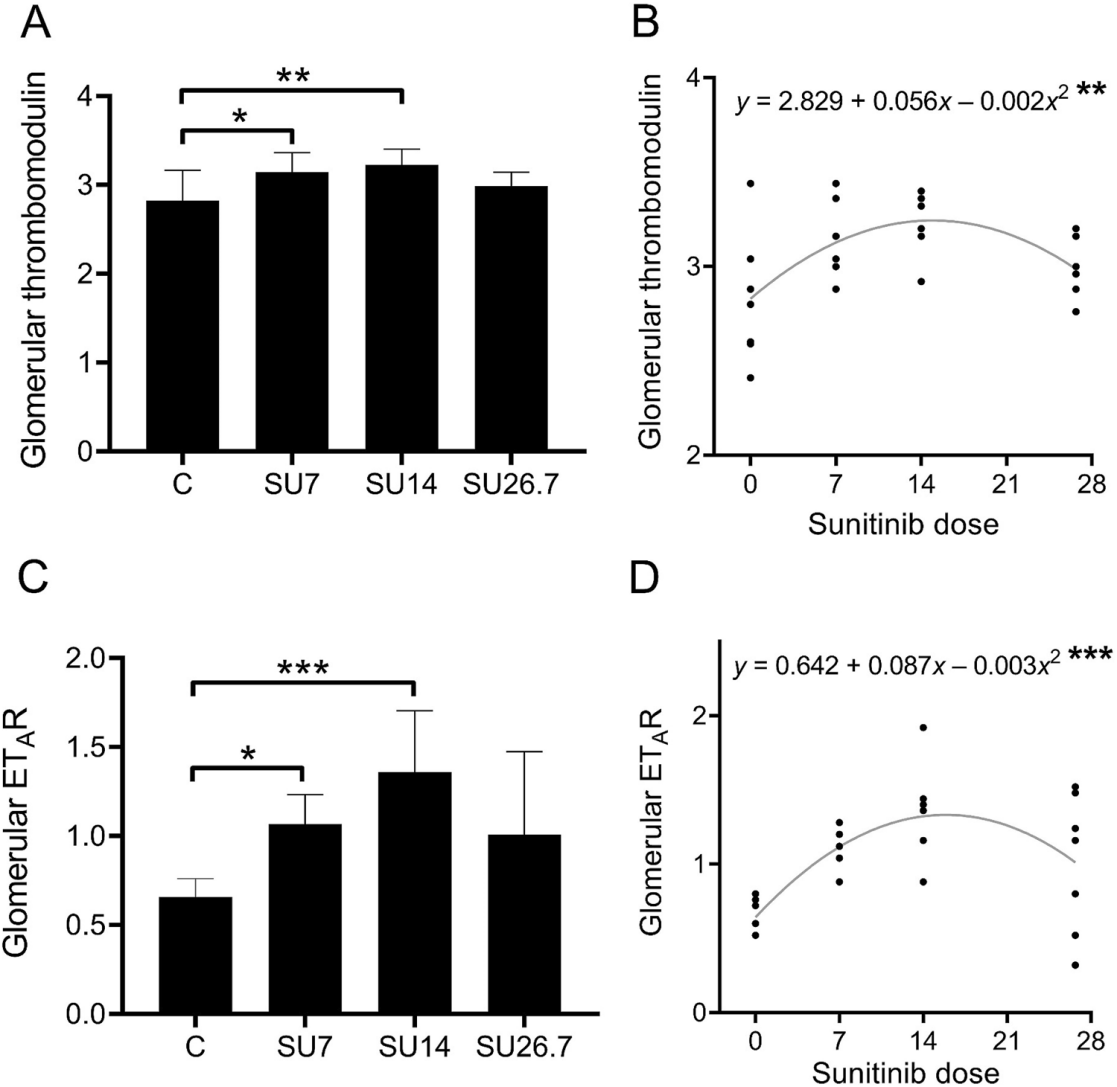
Glomerular thrombomodulin and ET_A_R are increased in rats exposed to a low and intermediate dose, but not a high dose of sunitinib. (**a**) Glomerular thrombomodulin protein was increased in rats exposed to a low and intermediate dose of sunitinib compared to controls, but not in kidneys of rats exposed to a high dose of sunitinib. (**b**) Glomerular thrombomodulin protein expression was associated with sunitinib dose. (**c**) Glomerular ET_A_R protein was increased in rats exposed to a low and intermediate dose of sunitinib compared to controls, but not in kidneys of rats exposed to a high dose of sunitinib. (**d**) Glomerular ET_A_R protein is non-linearly associated with the sunitinib dose. *p < 0.05, **p < 0.01, ***p < 0.001. SU, sunitinib; ET_A_R, endothelin A receptor.

None of the scored glomeruli in rats were negative for thrombomodulin. Glomerular thrombomodulin protein was increased in the kidneys of rats exposed to the low and intermediate doses of sunitinib (*p* < 0.05, Fig. 2A), but not in rats exposed to the highest dose of sunitinib. In support of these findings, a regression model showed that glomerular thrombomodulin protein levels were non-linearly associated with the sunitinib dose as a statistically significant quadratic relation (Fig. 2B , y = 2.829 + 0.056*x* − 0.002*x*^2^, *p* < 0.01).

To study the factors that may induce an upregulation of glomerular thrombomodulin, we next stained for the ET_A_R, which mediates kidney damage induced by VEGF inhibition. In rats treated with low and intermediate doses of sunitinib, but not in rats treated with a high dose of sunitinib, glomerular ET_A_R staining was increased compared to control rats (*p* < 0.01, Fig. 2C). In parallel with thrombomodulin levels, glomerular ET_A_R levels were quadratically associated with the sunitinib dose (*y* = 0.642 + 0.087*x* − 0.003*x*^2^, *p* < 0.001). Without taking into account the different treatment groups, glomerular ET_A_R protein levels were directly correlated with glomerular thrombomodulin protein levels (r: 0.460, *p* < 0.05).

### ***Treatment with the ET***_***A***_***R blocker sitaxentan normalises thrombomodulin expression***

To study whether thrombomodulin expression is regulated by endothelin signalling, rats exposed to the intermediate dose of sunitinib were treated with ETR blocking agents. Treatment with a selective ET_A_R antagonist (sitaxentan) normalised blood pressure and albuminuria in sunitinib-treated rats^14^ (Supplementary Figure S4). Conversely, treatment with a dual ET_A/B_R antagonist (macitentan) resulted in normalisation of blood pressure but not in the normalisation of sunitinib-induced albuminuria^14^ (Supplementary Figure S4).

**Figure 3:**
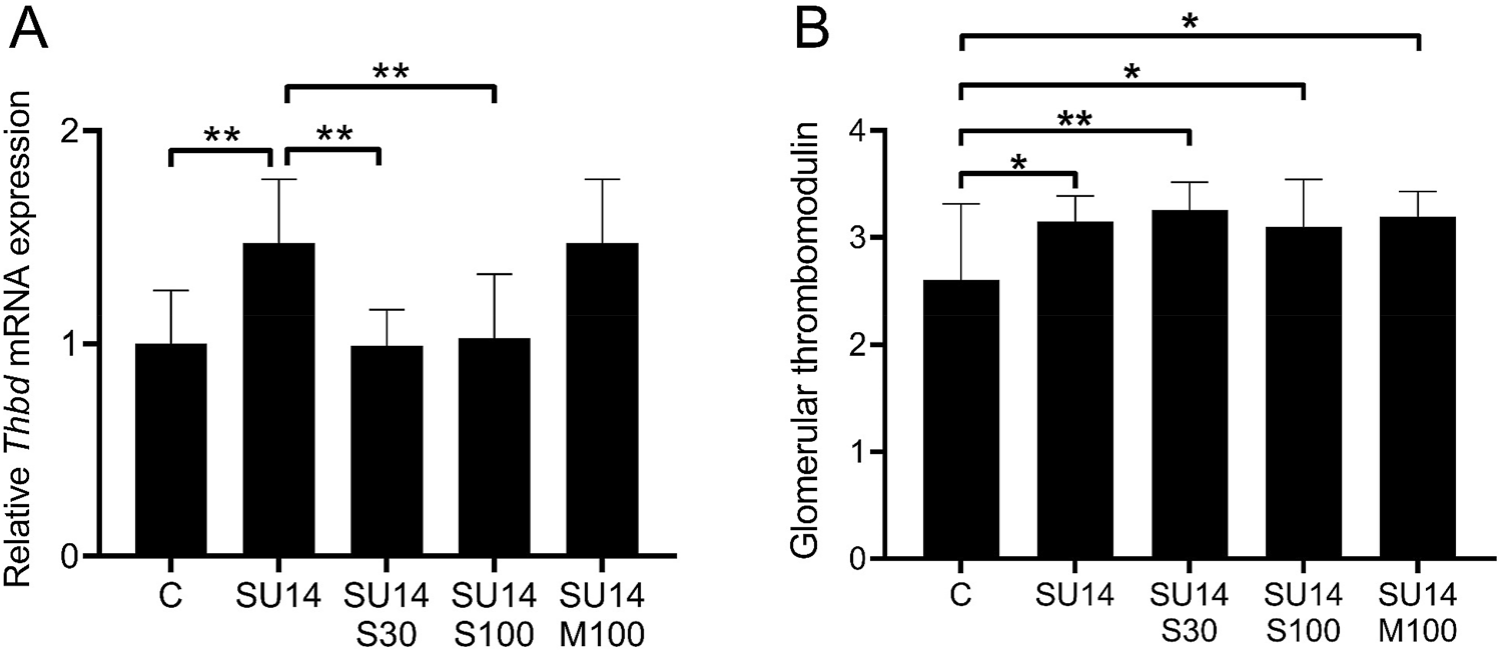
Renal thrombomodulin expression in rats exposed to sunitinib with sitaxentan or macitentan treatment. (**a**) Exposure to sunitinib results in a higher renal thrombomodulin mRNA expression, and sitaxentan (ET_A_R antagonist) at both dosages normalised this increase. (**b**) Sunitinib exposure resulted in a higher glomerular thrombomodulin protein expression compared to controls. Administration of sitaxentan (ET_A_R antagonist) nor macitentan (dual ET_A/B_R antagonist) resulted in a normalisation of the increased glomerular thrombomodulin protein expression. *p < 0.05, **p < 0.01.

Thrombomodulin mRNA expression was increased in the kidneys of rats exposed to sunitinib compared to controls (Fig. 3A , *p* < 0.01). Administration of sitaxentan, at both concentrations, on top of sunitinib normalised thrombomodulin mRNA expression to control levels (*p* < 0.01). However, administration of macitentan did not affect thrombomodulin mRNA levels. Relative thrombomodulin mRNA expression was directly associated with relative ET_A_R mRNA expression in the control group (r: 0.936, *p* < 0.01). In animals exposed to sunitinib only, thrombomodulin mRNA expression was directly associated with the amount of albuminuria (r: 0.876, *p* < 0.01).

At the protein level, sunitinib exposure resulted in higher glomerular thrombomodulin expression as compared to controls (Fig. 3B, *p* < 0.05). Neither co-administration of sunitinib with sitaxentan or macitentan resulted in the normalisation of the sunitinib-induced increase in glomerular thrombomodulin protein (Fig. 3B).

## Discussion

Here, we show that the nephroprotective protein thrombomodulin is increased in the kidney glomeruli of women with pre-eclampsia. Furthermore, we show that in rats exposed to the angiogenesis inhibitor sunitinib, glomerular thrombomodulin is upregulated in a dose-dependent manner, and the increase in glomerular thrombomodulin precedes the onset of histopathological manifestations. Finally, we show that blocking the ET_A_R normalises the sunitinib-induced upregulation of glomerular thrombomodulin. Our findings suggest that the upregulation of glomerular endothelial thrombomodulin occurs at an early stage of pre-eclamptic renal damage and serves as a mechanism to protect the glomerular filtration barrier.

Our study is the first to report increased glomerular thrombomodulin expression in pre-eclampsia. Since we previously found that in the placentas of women with pre-eclampsia, thrombomodulin is downregulated^24^, the increased serum levels of soluble thrombomodulin ectodomain in pre-eclampsia patients^17^ may be derived from the glomerular endothelium. Furthermore, we found an inverse association between glomerular thrombomodulin expression and the presence of glomerular endotheliosis, suggesting that the upregulation of thrombomodulin serves as a glomerular protection mechanism. This notion is supported by preclinical evidence in diabetic mice, in which thrombomodulin critically preserves glomerular cell survival and suppresses glomerular complement activation^19,20^. In contrast to our finding that an antiangiogenic state increases glomerular thrombomodulin levels, a previous study found that sFlt-1 treatment reduced glomerular thrombomodulin expression in rats with crescentic glomerulonephritis^25^. Hara et al.^25^ did not study the effect of sFlt-1 on glomerular thrombomodulin in healthy rats. Therefore, although increased levels of inflammatory factors are also found in pre-eclampsia, the downregulation of glomerular thrombomodulin in this study may be explained by the high-grade glomerular inflammation present in glomerulonephritis (proinflammatory cytokines, complement activation), which strongly suppress thrombomodulin expression^26^.

In order to elucidate whether in pre-eclampsia, glomerular thrombomodulin is upregulated as a result of systemic VEGF-receptor inhibition, we studied thrombomodulin expression in rats exposed to sunitinib. Sunitinib dose-dependently increased glomerular thrombomodulin at low and intermediate doses, but not at the highest dose. As histological changes were not seen in the kidneys of rats exposed to low-dose sunitinib^23^, the increase in glomerular thrombomodulin expression preceded the onset of glomerular histopathological damage induced by sunitinib. In rats exposed to an intermediate dose of sunitinib, the highest expression level of glomerular thrombomodulin was observed, and this was directly associated with the amount of albuminuria. The renal histopathological phenotype of our human pre-eclampsia cases was comparable to the renal damage of rats treated with an intermediate dose of sunitinib: endotheliosis was observed in 6 of 11 pre-eclampsia cases and microthrombi were observed in 1 of 11 pre-eclampsia cases^21^, whereas in the rat model, intermediate dose-treated rats had endotheliosis and the highest dose-treated rats had endotheliosis combined with fibrin deposits^23^. We propose that glomerular thrombomodulin expression increases at an early stage of pre-eclampsia and angiogenic inhibition, as a protective mechanism to offset antiangiogenic stress to the glomerular filtration barrier. In contrast, in the highest dose-treated rats, thrombomodulin expression was reduced to control levels. A recent study found that the dimensions of the endothelial glycocalyx layer are reduced in women with severe early onset pre-eclampsia^27^. Therefore, we also propose that in the high-dose sunitinib treated rats, the endothelial glycocalyx is affected, which elicits interaction between thrombomodulin and enzymes, thereby contributing to the proteolytic cleavage and loss of glomerular thrombomodulin protein.

In our pre-eclampsia cases, the increased glomerular thrombomodulin levels were associated with less glomerular endotheliosis, a lesion directly caused by perturbed glomerular VEGF signalling. In vitro studies have identified VEGF as a potent inducing factor of thrombomodulin expression in endothelial cells^28^. Hence, the upregulation of renal thrombomodulin under antiangiogenic conditions such as pre-eclampsia and angiogenic inhibition may seem counterintuitive. Interestingly, one study showed that VEGF is increased in the glomeruli of women with pre-eclampsia^29^, likely reflecting a glomerular compensatory mechanism in an attempt to offset the reduced VEGF bioavailability. Thus, the upregulation of thrombomodulin may be caused by a compensatory increase of glomerular VEGF under systemic antiangiogenic conditions. Furthermore, endothelin signalling may be involved in the regulation of glomerular thrombomodulin. Plasma endothelin levels are increased in women with pre-eclampsia^30^, and are associated with systemic sFlt-1 levels^31,32^. In our rat model, both treatment with sitaxentan (ET_A_R antagonist) and macitentan (dual ET_A/B_R antagonist) resulted in normalisation of sunitinib-induced hypertension in our rats^14^. However, only treatment with sitaxentan (ET_A_R antagonist) normalised the sunitinib-induced upregulation of renal thrombomodulin and decreased albuminuria. This suggests that regulation of renal thrombomodulin expression is independent of hypertension-mediated endothelial damage, which was also supported by the lack of increased thrombomodulin expression in our hypertensive control patients. It remains to be investigated whether the ET_A_R induces thrombomodulin expression via systemic effects or via signalling in glomerular endothelial cells specifically. Interestingly, thrombomodulin expression was only normalised at the mRNA level after ET_A_R blockade in sunitinib-treated rats, but not at the protein level. This discrepancy may be explained by post-translational effects, half-life of the protein and factors that influence the cleavage of thrombomodulin from the endothelium.

Thrombomodulin has important anti-inflammatory properties, that include the inhibition of leukocyte infiltration by sequestering proinflammatory molecules such as the Lewis y antigen and HMGB-1^33,34^, decreasing NF-κB activity^35^ and inhibiting complement activation^20^. Previously, we reported that in our pre-eclampsia cases, glomerular complement activation was increased compared to pregnant and hypertensive controls^36^. This suggests that the upregulation of glomerular endothelial thrombomodulin in pre-eclampsia functions as an anti-inflammatory and anti-complement mechanism. In addition to its well-known anti-inflammatory effects, a recent study found that thrombomodulin has proinflammatory properties; thrombomodulin binds the leukocyte integrins LFA-1 and Mac1, thereby promoting leukocyte adhesion to the endothelium in vitro^37^. The increase in glomerular thrombomodulin may therefore also directly contribute to glomerular inflammation and damage.

We must acknowledge the limitations of our study. Firstly, the women with pre-eclampsia in whom we investigated glomerular thrombomodulin expression all died because of the consequences of pre-eclampsia. This makes these women a specific subgroup of pre-eclampsia patients and therefore the results of this study may not be applicable to all women who develop pre-eclampsia. Secondly, male rats treated with sunitinib were used as a model to study thrombomodulin regulation under antiangiogenic conditions. Moreover, sunitinib leads to inhibition of diverse receptor tyrosine kinases and is not a specific inhibitor of VEGF. Clearly, this model does not fully mimic pre-eclampsia, however, sunitinib treatment does lead to hypertension and proteinuria^23^. Therefore, we were able to use this model to study thrombomodulin regulation under antiangiogenic conditions. Despite the limitations of our study, it puts forward interesting insights into the mechanisms of renal damage in pre-eclampsia.

In conclusion, renal thrombomodulin expression is increased in pre-eclampsia and in a sunitinib-induced model of angiogenic inhibition. We speculate that the increase in glomerular thrombomodulin levels reflects a reno-protective mechanism in response to antiangiogenic stress to the glomerular filtration barrier. Furthermore, treatment with an ET_A_R antagonist normalised renal thrombomodulin mRNA expression concomitantly with reducing proteinuria in sunitinib-treated rats. More research is warranted on the role of thrombomodulin in the glomerular filtration barrier.

## Supplementary Information

Supplementary Information
