# Supplementary material for: Thrombomodulin is upregulated in the kidneys of women with pre-eclampsia
Source: Sci Rep. 2021 Mar 11;11:5692. doi: 10.1038/s41598-021-85040-9 (PMC7952396; doi:10.1038/s41598-021-85040-9)
Supplement: Supplementary file 1 — Supplementary Information [file 41598_2021_85040_MOESM1_ESM.pdf]

Thrombomodulin is upregulated in the kidneys of women with pre-eclampsia

Cleo C.L. van Aanhold [1*], Manon Bos [1,2], Katrina M. Mirabito Colafella [3,4], Marie-Louise P. van der Hoorn [2], Ron Wolterbeek [1], Jan A. Bruijn [1], Kitty W.M. Bloemenkamp [5], Anton H. van den Meiracker [3], A.H. Jan Danser [3] and Hans J. Baelde [1]

[1] Department of Pathology, Leiden University Medical Center, The Netherlands;
[2] Department of Obstetrics and Gynaecology, Leiden University Medical Center, The Netherlands;
[3] Department of Internal Medicine, Erasmus Medical Center, The Netherlands;
[4] Cardiovascular Disease Program, Biomedicine Discovery Institute and Department of Physiology, Monash University, Australia;
[5] Department of Obstetrics, Birth Center, University Medical Center Utrecht, The Netherlands

RUNNING HEAD: Glomerular thrombomodulin in pre-eclampsia

Correspondence:

Cleo van Aanhold (C.C.L.van_Aanhold@lumc.nl)

Supplementary material

Supplementary Table S1. Primer sequences used for quantitative PCR

Gene	Primer sequence (F: Forward, R: Reverse)	
Hprt	F	GGC TAT AAG TTC TTT GCT GAC CTG
	R	AAC TTT TAT GTC CCC CGT TGA
Thbd	F	CCT TTG TCT TTC CGG GCT CT
	R	TCA AGT CCT CCC TAC CCT CG
Ednra	F	AGG GGA TCC CGA TTC CTT GA
	R	GTG GGC ATC ACT GTC CTG AA

Supplementary Table S2: Clinical and histological characteristics of the autopsy cohort.

Characteristic	PE (n=11)	PC (n=22)	HC (n=11)	*P*-value
Age, years	32.2±5.5	31.6±5.2	37.3±7.7	0.038
Gestational age, wk	36.0±3.1	29.7±12.3	NA	0.031
Parity	0.6±1.0	0.7±0.9	NA	0.743
Proteinuria, g/24h	3.5±6.4	ND	ND	
Systolic pressure, mmHg	165±24	124±14	ND	<0.001
Diastolic pressure, mmHg	106±16	82±15	ND	0.006
Death-autopsy interval, h	23.1±21.9	25.2±14.5	40.8±33.6	0.134
Histology, n (%)				
Acute tubular necrosis	0 (0)	4 (18)	2 (18)	0.314
Endotheliosis				0.030
None	5 (46)	19 (86)	9 (82)	
<20% of the lumen	1 (9)	3 (14)	1 (9)	
20-80% of the lumen	3 (27)	0 (0)	1 (9)	
>80% of the lumen	2 (18)	0 (0)	0 (0)	
FSGS	1 (9)	2 (9)	4 (36)	0.101
Global sclerosis >1%	1 (9)	0 (0)	3 (27)	0.122
Glomerulitis	0 (0)	5 (23)	1 (9)	0.176
Hyalinosis	1 (9)	4 (18)	6 (55)	0.028
IFTA	0 (0)	0 (0)	1 (9)	0.215
Intima fibrosis	2 (18)	7 (32)	8 (73)	0.021
Ischemia	0 (0)	1 (5)	2 (18)	0.200
Mesangium changes	2 (18)	0 (0)	2 (18)	0.140
Microthrombi	1 (9)	0 (0)	1 (9)	0.351
Edema	1 (9)	0 (0)	0 (0)	0.215
Podocyte changes	2 (18)	0 (0)	0 (0)	0.043
Tram tracking	4 (36)	0 (0)	0 (0)	<0.001

Data are presented as the mean ± standard deviation, unless stated otherwise. FSGS, focal segmental glomerulosclerosis; IFTA, interstitial fibrosis and tubular atrophy; NA, not applicable; ND, not determined.

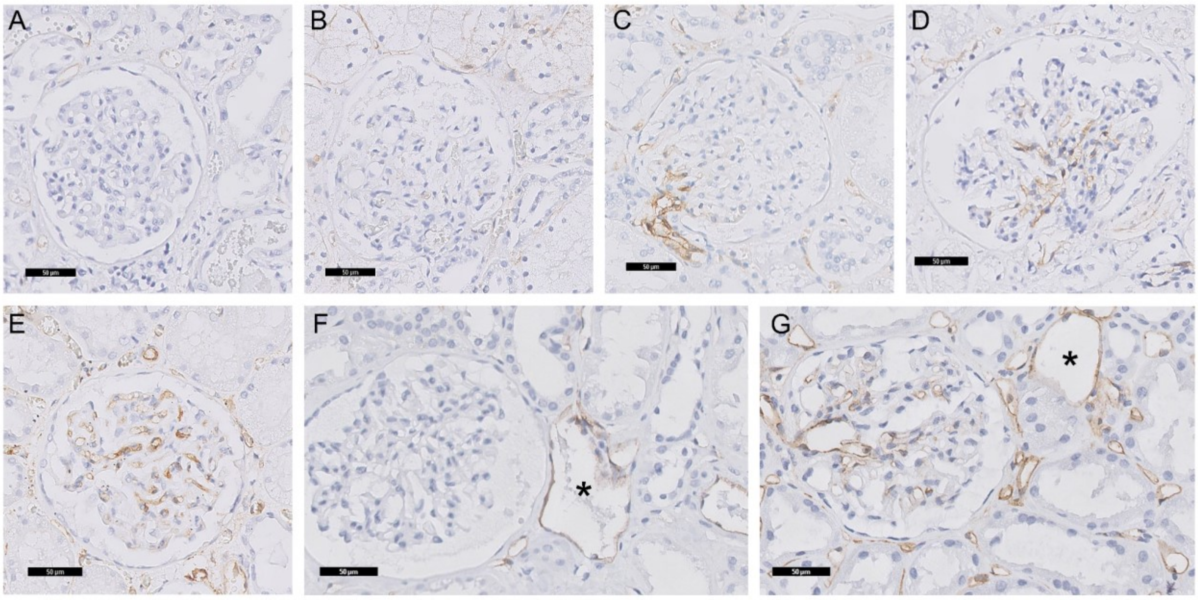

Supplementary Figure S1. The distribution of glomerular thrombomodulin in the autopsy cohort. **(a)** Representative example of a glomerulus in which thrombomodulin was absent. In the peritubular capillaries, thrombomodulin was present; **(b)** Representative example of a glomerulus in which less than 10% of the glomerular capillaries were positive for thrombomodulin; **(c)** Representative example of a glomerulus in which 10-50% of the glomerulus was positive for thrombomodulin; **(d)** Representative example of a glomerulus in which 50-90% of the glomerulus was positive for thrombomodulin; **(e)** Representative example of a glomerulus in which more than 90% of the glomerulus was positive for thrombomodulin; **(f)** Representative example of peritubular thrombomodulin staining surrounding a glomerulus in which thrombomodulin is absent; **(g)** Representative example of peritubular thrombomodulin staining (asterisk) surrounding a glomerulus in which thrombomodulin is present (asterisk).

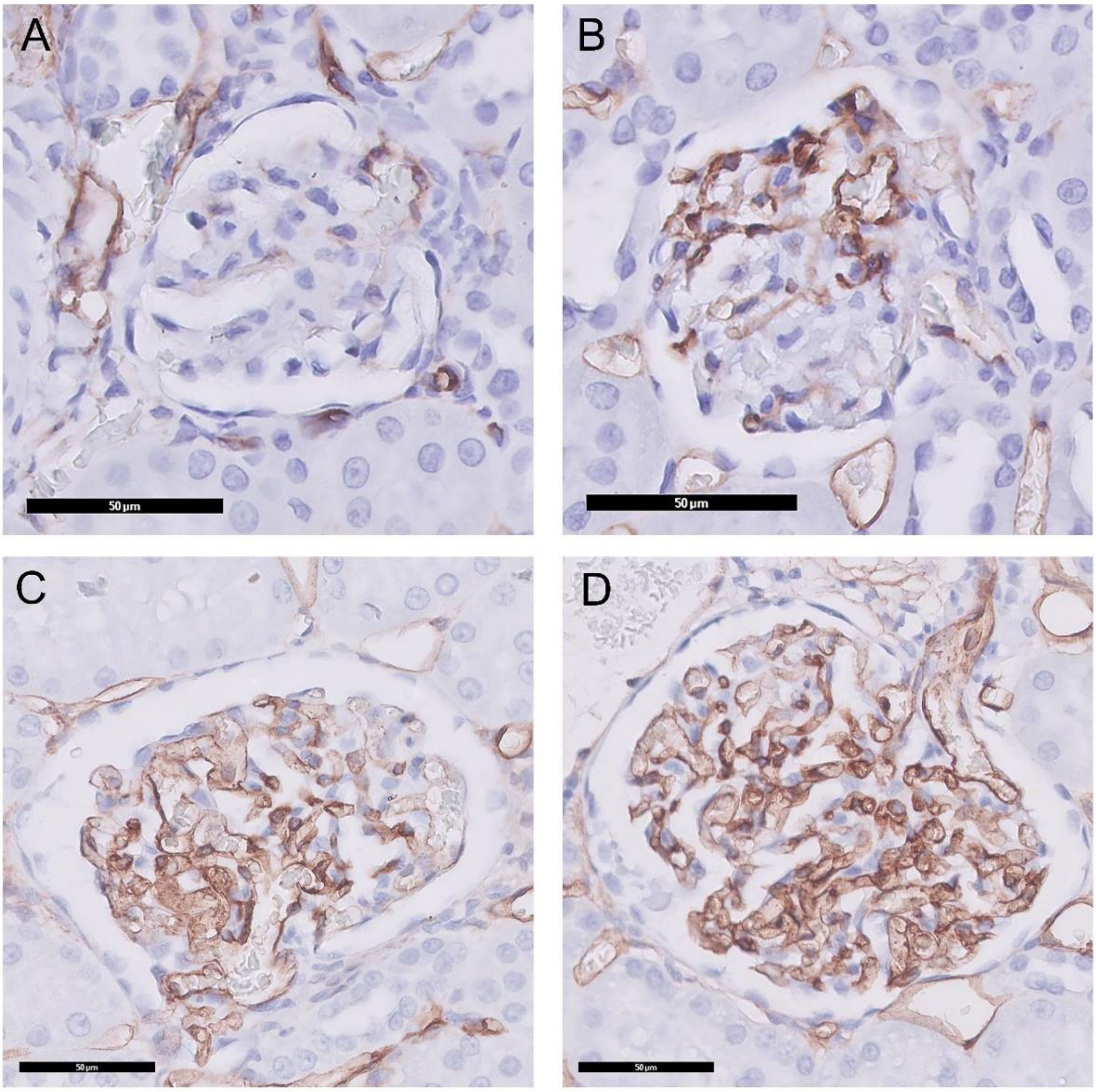

Supplementary Figure S2. The distribution of glomerular thrombomodulin in rats. (a) Representative example of a glomerulus in which less than 10% of the glomerular capillaries were positive for thrombomodulin; (b) Representative example of a glomerulus in which 10-50% of the glomerulus was positive for thrombomodulin; (c) Representative example of a glomerulus in which 50-90% of the glomerulus was positive for thrombomodulin; (d) Representative example of a glomerulus in which more than 90% of the glomerulus was positive for thrombomodulin.

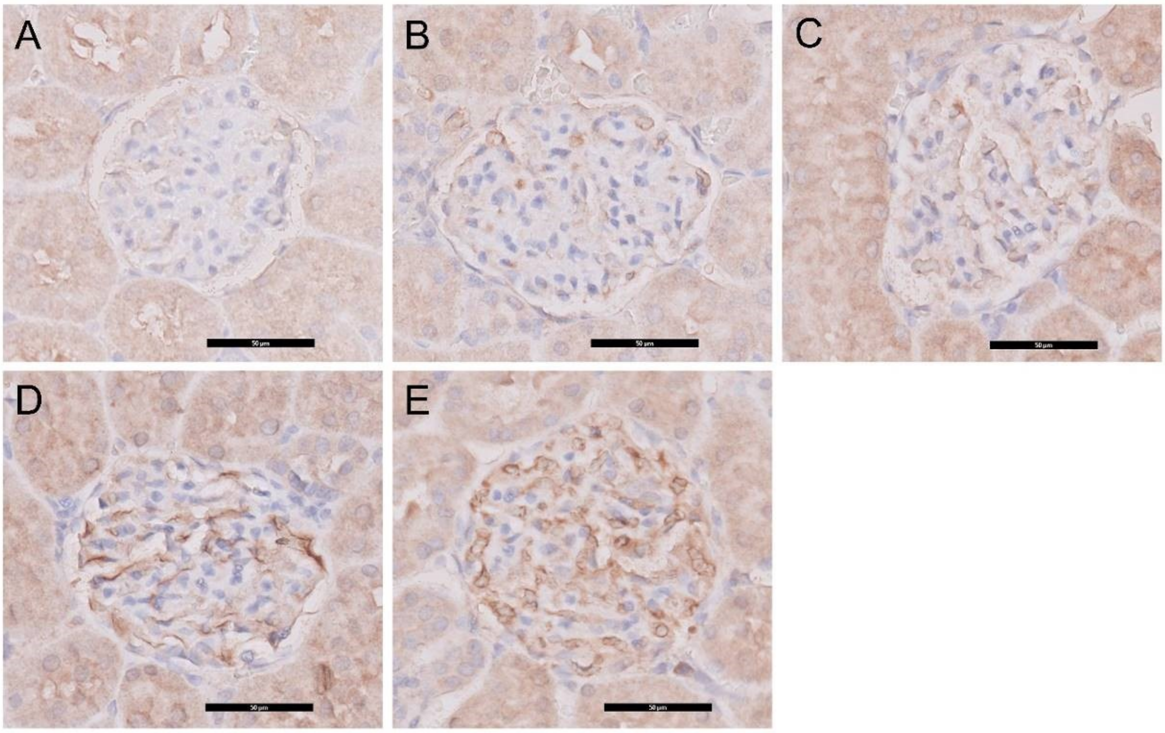

Supplementary Figure S3. The distribution of glomerular ET_AR in rats. (a) Representative example of a glomerulus in which less than 10% of the glomerulus was positive for ET_AR; (b) Representative example of a glomerulus in which 10-50% of the glomerulus was positive for ET_AR; (c) Representative example of a glomerulus in which 50-90% of the glomerulus was positive for thrombomodulin; (d) Representative example of a glomerulus in which more than 90% of the glomerulus was positive for thrombomodulin.

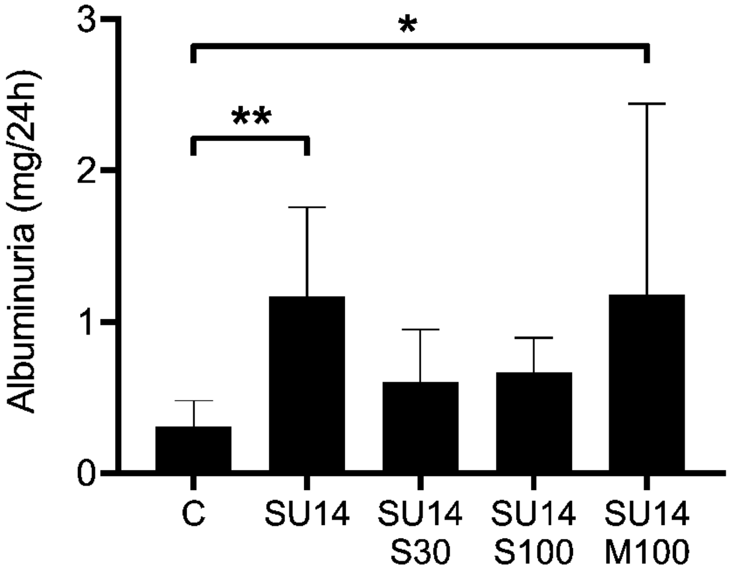

Supplemental Figure S4. Albuminuria in rats exposed to sunitinib with sitaxentan or macitentan treatment. Sunitinib exposure resulted in albuminuria. Co-treatment with sitaxentan (ET_AR antagonist) at both dosages reduced albuminuria; co-treatment with macitentan (dual $ET_{A/B}R$ antagonist) did not reduce albuminuria. *$p<0.05$, **$p<0.01$.
